# Validation of a discovery MI 4-ring model according to the NEMA NU 2-2018 standards: from Monte Carlo simulations to clinical-like reconstructions

**DOI:** 10.1186/s40658-024-00616-4

**Published:** 2024-01-31

**Authors:** Antoine Merlet, Benoît Presles, Kuan-Hao Su, Julien Salvadori, Farzam Sayah, Hanieh Jozi, Alexandre Cochet, Jean-Marc Vrigneaud

**Affiliations:** 1 Imagerie et Vision artificielle, ImViA EA 7535, University of Burgundy, Dijon, France; 2 Institut de Chimie Moléculaire de l’Université de Bourgogne (ICMUB), UMR CNRS 6302, University of Burgundy, Dijon, France; 3 GE Healthcare, Waukesha, WI USA; 4 ICANS, Institut de cancérologie Strasbourg Europe, Strasbourg, France; 5 Department of Nuclear Medicine, Georges-François Leclerc Cancer Centre, Dijon, France

**Keywords:** PET, Nuclear medicine, GATE, Monte Carlo simulation, NEMA

## Abstract

**Background:**

We propose a comprehensive evaluation of a Discovery MI 4-ring (DMI) model, using a Monte Carlo simulator (GATE) and a clinical reconstruction software package (PET toolbox). The following performance characteristics were compared with actual measurements according to NEMA NU 2-2018 guidelines: system sensitivity, count losses and scatter fraction (SF), coincidence time resolution (CTR), spatial resolution (SR), and image quality (IQ). For SR and IQ tests, reconstruction of time-of-flight (TOF) simulated data was performed using the manufacturer’s reconstruction software.

**Results:**

Simulated prompt, random, true, scatter and noise equivalent count rates closely matched the experimental rates with maximum relative differences of 1.6%, 5.3%, 7.8%, 6.6%, and 16.5%, respectively, in a clinical range of less than 10 kBq/mL. A 3.6% maximum relative difference was found between experimental and simulated sensitivities. The simulated spatial resolution was better than the experimental one. Simulated image quality metrics were relatively close to the experimental results.

**Conclusions:**

The current model is able to reproduce the behaviour of the DMI count rates in the clinical range and generate clinical-like images with a reasonable match in terms of contrast and noise.

## Background

Positron emission tomography (PET)/computed tomography (CT) is a well-established molecular imaging technique for cancer diagnosis and treatment response monitoring [1]. Its use in clinical routine has increased steadily in recent years and, and at the same time, its performance has never stopped evolving over time. Recent developments such as time-of-flight (TOF), point spread function modelling (PSF), digital PET detectors, and long axial field-of-view (FOV) have taken the imaging capabilities of the technique even further [2, 3].

The use of simulations in PET has long been recognised as a valuable tool for a number of applications, including detector design, evaluation of image reconstruction algorithms, correction techniques, dosimetry, and pharmacokinetic modelling [4]. In emission tomography, simulations can be useful to study the impact of different parameters (acquisition, reconstruction, corrections, etc.) on the quality of PET images with more flexibility than what could be achieved with physical phantoms [5], while requiring fewer resources and less expense.

There are several PET imaging simulators in the scientific community that differ in their level of complexity and computational resource requirements (for a complete review, see [6, 7]). Monte Carlo-based (MC) simulators are usually considered the gold standard as they can adequately model the physical processes that occur during radiation transport in media [8]. Among the MC simulators, Geant4 Application for Tomographic Emission (GATE) [9] is a well-known simulation toolkit, historically developed for nuclear imaging with specific layers for modelling sources, detection geometries, and detector electronic responses. GATE has been successfully used to validate the performance of several existing PET systems or to study the impact of different detector designs [7, 10–18].

To validate the modelling of a clinical PET scanner, the simulated data are usually compared with the experimental data according to the National Electrical Manufacturers Association (NEMA) standards [19]. In its latest version, this standard codifies seven tests used to characterise a PET system, and four of them are commonly used in the literature to validate MC models: the spatial resolution, the sensitivity, the count losses and scatter fraction, and the image quality [7, 12, 17]. For the spatial resolution and image quality tests, the PET data must be reconstructed. As commercial reconstruction software are not designed nor adapted to reconstruct simulated data, MC simulators are often used in conjunction with open-source third-party software, such as STIR [20] or CASToR [21]. While such software is flexible, they generally require a system-specific implementation of the corrections used in the reconstruction. This implementation is complex, and the reconstructed images may differ from images obtained with the manufacturer’s reconstruction tools, which are optimised for their own scanner. However, because reconstruction is a specific field in itself, validation of simulated systems is often limited to data acquisition, and reconstruction tests are ignored or bypassed [11, 12, 15, 16, 22, 23].

Regarding the validation of MC models of recent digital PET systems, the Vereos machine (Philips Medical Systems, Eindhoven, The Netherlands) has been studied extensively by Salvadori et al. [7]. The Vision PET/CT system (Siemens, Erlangen, Germany), for its part, has been modelled by Zein et al. [24] in the context of a hypothetical model with sparse detector module rings and extended axial field of view. MC models of the Discovery MI (GE HealthCare, Chicago, Il, USA) have been investigated by two groups [23, 25]. Tiwari et al. [23] have used a GATE model to predict the performance of the system with extended axial FOVs. Kalaitzidis et al. [25] have worked on a pipeline to reconstruct PET images from data simulated in GATE using CASToR. None of these works provide a complete investigation of their models as the former did not study the reconstructed image quality, and the latter did not model the behaviour of the DMI at very high count rates.

In this work, we propose a complete validation of a GATE model of the DMI 4-ring system, performed against experimental measurements, using the latest NEMA standards [19]. In addition, reconstructions of the simulated and experimental data were performed using the manufacturer’s reconstruction software, allowing the same clinical-like corrections to be applied to both datasets.

## Materials and methods

### Scanner geometry

**Fig. 1:**
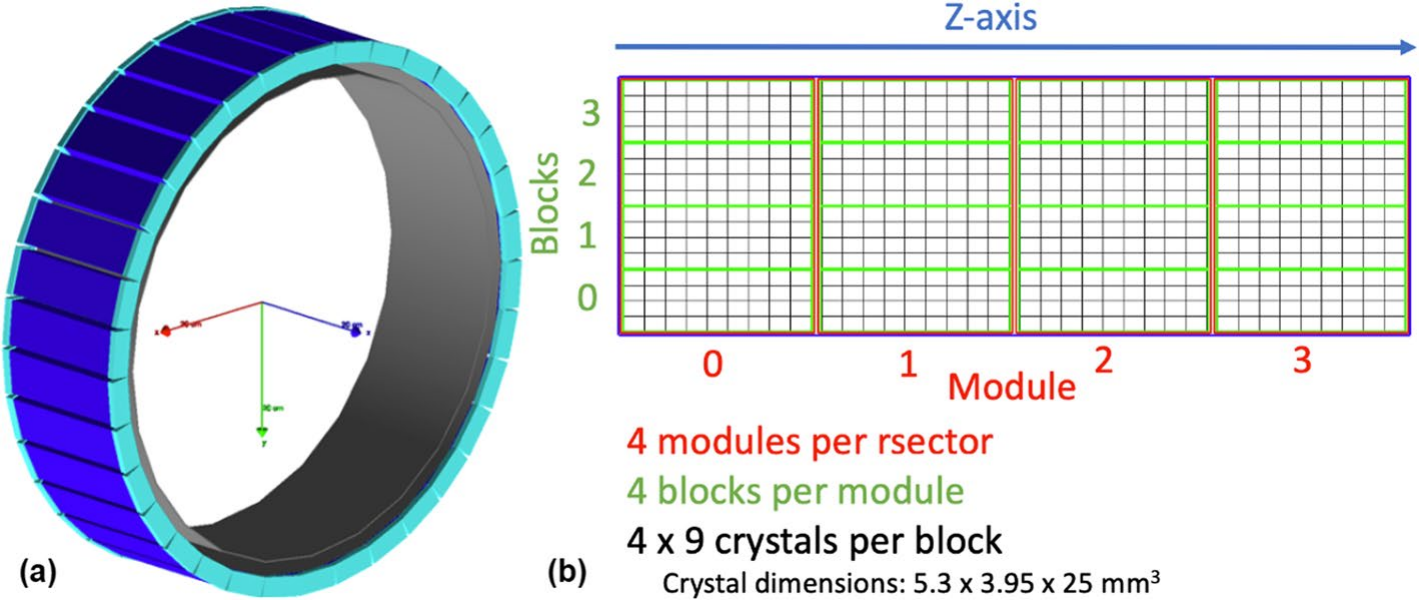
**a** GATE representation of the DMI 4-ring with the *rsectors* (34 in totals) in blue, the lead shielding in cyan and the detector covers in grey and **b** the structure of a *rsector*

The DMI is a silicon photomultiplier-based PET/CT scanner, with lutetium-yttrium oxyorthosilicate (LYSO) crystals. Partial description of the geometry of different DMI PET systems have already been reported [26–29]. In this work, the geometry of the DMI 4-ring (including crystal/module spacing) and the detectors configuration (indexing) have been modelled according to the data provided by the manufacturer. In GATE, the *rsector* is the largest geometry inside the *cylindricalPET*, and a total of 34 *rsectors* were arranged to form the scanner ring. Each *rsector* contained four *modules*, adjacent in the axial direction. In each *module*, four *blocks* were stacked, containing a $4 \times 9$ array of LYSO *crystals*. The *crystals* dimensions were 5.3 (axial) $\times ~3.95$ (transaxial) $\times ~25$ (length) mm$^3$. Overall, this geometry gave a 20-cm axial FOV and a 70-cm transaxial FOV. The *rsectors’* lead shielding and the inner plastic cover were also modelled. Figure 1 shows the visual representation of the DMI model in GATE, without the patient bed. When the patient bed was used in experimental acquisitions, it was also included in the associated GATE simulations.

### Simulation environment/framework

GATE 9.0 and Geant4 10.5 were used to model the DMI. The GATE physics list was set to $emstandard\_ opt{4}$ and no custom cuts or variance reduction techniques were used, i.e. all particles were generated and tracked according to the default behaviour defined in Geant4. The radioactive source was set to $\beta +$ emissions for all simulations, where *setForcedUnstableFlag* was used to ensure source decay with *setForcedHalfLife* 6586.2 s. The $^{18}F$ branching ratio to $\beta +$ was reproduced by multiplying the desired ^18^F source activity by 0.969 in the GATE simulation. The energy of the $\beta +$ source was set according to the Landolt-Börnstein tables available in GATE (*energytype Fluor18*). The simulations were performed on the Centre de Calcul de l’Université de Bourgogne (CCUB) using computers with Intel Xeon Gold CPU 6126 @ 2.60 GHz and 64 GB memory. To speed up GATE simulations, each simulation was equally divided into *n* sub-simulations with $t_{subsimulation} = t_{simulation}/n$; where $n\in \mathbb {N^*}$, *t*_subsimulation_ is the simulation time of each sub-simulation and *t*_simulation_ is the simulation time of the whole GATE simulation. Our simulations were performed with $200\le n \le 400$, depending on the expected simulation time.

All NEMA tests were analysed using in-house tools implemented in Python/C++. They were validated by comparing the experimental results processed using these tools with those obtained using the manufacturer’s software tools (see Appendix “Simulation environment/framework”). However, as the time-of-flight resolution test was not available in the manufacturer’s software for our Generation 1 (Gen1) DMI 4-ring, the results of our in-house tool were compared with published data obtained from a Generation 2 (Gen2) DMI 6-ring (see “Time-of-flight resolution”).

### Data processing

When performing acquisitions on the DMI, experimental data could either be stored in list mode or in three-dimensional (3D) sinograms. The list mode data could then be rearranged in 3D sinograms using a proprietary offline reconstruction package, hereafter referred to as the PET toolbox (GE HealthCare, Chicago, Il, USA). The output of a GATE simulation provides a list mode with single and coincidence events, stored as a Python NumPy array. This list mode includes the exact position of the annihilations, and the type of each coincidence (i.e. true, random, or scatter). Simulated list modes were organised into 3D sinograms using GATE detector numbers and look-up tables provided by the manufacturer. The 3D sinograms were of dimensions 415 (radial bins) × 1261 (planes) × 272  (projections). The TOF sinograms were of dimensions 1261 (planes) × 29 (time) × 415 (radial bins) × 272 (projections).

### Image reconstruction

Image reconstruction of clinical data was done using the PET toolbox. It includes reconstruction algorithms such as filtered back-projection (FBP) and ordered-subsets expectation-maximisation (OSEM). Standard correction methods (implemented by the manufacturer) are provided within the PET toolbox: normalisation, decay, well-counter (calibration for quantification), attenuation, deadtime, random and scatter corrections [30–33].

In order to reconstruct simulated data, a specific interface has been added to the PET toolbox, allowing corrections for normalisation, attenuation, randoms and scatters. Currently, it does not support corrections for decay, deadtime and well-counter. This interface requires not only the sinograms to be reconstructed, but also calibration files for normalisation correction (geometrical factors and individual detector efficiencies), and an attenuation map for attenuation correction. The normalisation correction was performed according to a component-based method [34, 35]. To determine geometrical factors (each detector has the same detection efficiency in the model), two simulations were run at very high statistics where only true coincidences were recorded. The first simulation was performed with an annulus source of 32 cm radius, of 1 mm thickness, and 20 cm long, centred on the FOV of the scanner, where 3.2 billion true coincidences were collected over 253 CPU days of simulation. The normalisation factors were calculated with the help of a second simulation, using a flood source of 10 cm radius and 22 cm height positioned at the centre of the FOV, where 562 million of true counts were collected. This simulation took 137 CPU days to complete. The simulated attenuation map was generated in GATE using the *MuMap actor*. The output image contained the spatial distribution of the linear attenuation coefficient at 511 keV for the given study. Its dimensions and resolution were 256 × 256 × 71 pixels and 2 × 2 × 3 mm^3^, respectively. For the DMI, the estimation of random coincidences is performed on single events [30]. The proposed randoms from singles (RFS) formula shown in Eq. 1 has been applied to estimate random counts of the simulated data. For two given detectors *x* and *y* and their associated singles count rates $s_x$ and $s_y$, the estimated randoms rate between the two detectors $r_{xy}$ is:

1 $$\begin{aligned} r_{xy}=(2\tau )s_xs_y \end{aligned}$$

where $2\tau$ is the coincidence window size. Finally, scatter correction was performed using a model-based algorithm as implemented in the PET toolbox for clinical data  [32]. Overall, the proposed methodology allows a close comparison between experimental and simulated data, as the sinograms have the same properties and the reconstruction algorithms and corrections used were similar to the clinical setup.

### NEMA studies

In this work, the NEMA NU 2-2018 [19] tests performed experimentally on the DMI scanner were compared with those simulated in GATE with the corresponding DMI model.

#### Sensitivity

A 700 mm-long, 1 mm inner diameter line source inserted into a 1.25-mm thick aluminium sleeve was filled with 3.3 MBq of ^18^F-FDG at the beginning of the acquisition and centred in the FOV. Five 60-second frames were acquired, successively increasing the number of aluminium sleeves around the source. Five more frames were acquired with the line source located at a radial offset of 10 cm from the centre. Simulations were strictly mimicking the experimental conditions. In both cases, 3D sinograms were rebinned into 2D sinograms using single-slice rebinning (SSRB) [36] and processed according to NEMA specifications. The system sensitivity (counts/sec/MBq) and the axial sensitivity profile were reported.

#### Scatter fraction, count losses, and randoms

Noise equivalent counts (NEC) and scatter fraction (SF) were computed according to NEMA using a ^18^F-FDG line source inserted into a cylindrical polyethylene phantom of 700 mm length and 101.5 mm radius. The line source had an inner diameter of 3.2 mm and a length of 700 mm. The activity at the start of the acquisition was 719 MBq. This phantom was built geometrically in GATE following the NEMA specifications, and the patient bed was also modelled.

Experimentally, 24 frames were acquired during ten hours: 17 contiguous acquisitions of 15 min, followed by seven acquisitions of 25 min, spaced 25 min apart. These long acquisitions, coupled with very high activity, could not be fully simulated in GATE, because the CPU time and computing power required would have been excessive. Therefore, the duration of the simulated acquisitions were set to obtain at least ten million prompts per frame, while maintaining the same number of frames and average activities.

Experimental and simulated data were rebinned into two-dimensional (2D) sinograms using SSRB and processed according to NEMA specifications.

#### Spatial resolution

The NEMA spatial resolution test was acquired on the DMI using two sets of three ^18^F-FDG point sources placed at 1, 10, and 20 cm from the FOV centre in the radial direction. One set was located in the central transverse plane of the scanner and the other at an offset of three-eighths of the axial FOV (i.e. 76 mm) from the FOV centre. In GATE, spheres of 0.5 mm radius were used to simulate the six point sources. The duration of the experimental and simulated acquisition was 60 s.

In both cases, the image reconstruction was performed using the same implementation of the FBP algorithm with a ramp filter and a cut-off at the Nyquist frequency (no smoothing). The reconstruction was centred on the sources with a transverse FOV of 250 mm so as to reach a voxel size of 0.65 × 0.65 × 2.79 mm^3^.

#### Time-of-flight resolution

For this test, the experimental and simulated data acquired during the count losses test (see “Scatter fraction, count losses, and randoms”) were used.

On DMI 4-ring Gen1 systems, the detection of coincidences is performed with a time resolution sampling *S* of 13.02 ps, but the obtained TOF list mode data are further mashed by a factor *C* of 13, resulting in a final sampling resolution of $CS = 169.26$ ps. Although this compression does not penalise the reconstruction of TOF data (the Nyquist–Shannon sampling theorem is respected), it has a major impact when using the NEMA method to evaluate the TOF resolution.

To process these mashed data, each 169.26 ps bin was uniformly resampled over 13 bins of 13.02 ps, and the TOF resolution of the system CTR_mash_ was estimated according to the NEMA guidelines. To account for the degradation of the CTR due to mashing, an empirical correction factor was then applied to obtain the final result CTR_cor_, assuming Gaussian-distributed data:

2 $$\begin{aligned} {\text {CTR}}_{\rm cor} = \sqrt{{\text {CTR}}_{\rm mash}^{2} - \left[ 2\sqrt{2 {\text{ln}}(2)} \times \frac{ CS}{\sqrt{6}}\right] ^2} \end{aligned}$$

In this expression, the term in square brackets represents the uncertainty contribution induced by the use of mashed data in the NEMA process:

- $2\sqrt{2 ln(2)}$ is the term used to obtain a Gaussian full width at half maximum (FWHM) from its standard deviation,
- $CS/\sqrt{6}$ is the derived global standard deviation of the uncertainty introduced by the mashing and the processing steps. It is the quadratic sum of the errors associated with (i) the mashing of a uniformly sampled distribution [0, *CS*] by a factor *C*, ($CS / \sqrt{12}$), and (ii) the uniform up-sampling from *CS*-sized bins to *S*-sized bins ($CS / \sqrt{12}$).

The same process was applied to the simulated data: the raw TOF data from GATE were mashed over 169.26 ps bins, then processed according to the NEMA guidelines to obtain the simulated CTR_mash_ and the simulated CTR_cor_ according to Eq. 2.

To determine the line source position, the experimental data were reconstructed using OSEM with all available corrections except decay correction. A 3D line was fitted to the centroid of each reconstructed axial plane, and the associated unit vector was computed using the intersection of this fit with the first and last axial planes. The simulated line source unit vector was determined based on the position of the phantom in GATE.

The timing error analysis was performed to obtain a TOF offset profile for experimental and simulated data by processing 20 million and 10 million counts, respectively. The TOF offset profile was corrected for scatters and randoms according to the NEMA report.

#### Image quality, accuracy of corrections

The image quality test was performed on the DMI with an IEC NEMA body phantom which contained 5.3 kBq/mL and 20.9 kBq/mL of ^18^F-FDG in the background and spheres, respectively, resulting in a 3.9:1 sphere-to-background ratio (SBR). The body phantom was centred in the FOV so that the middle of the spheres was at the centre of the axial FOV. According to NEMA, the NEC phantom (see “Scatter fraction, count losses, and randoms”) was placed on the patient bed outside the imaging FOV, aligned with the body phantom, and the line source was filled with approximately 120 MBq of ^18^F-FDG. Three repeated acquisitions were launched with decay-adjusted times of acquisition. Image quality analysis was performed according to NEMA for these three acquisitions and then averaged to obtain the final experimental results.

In GATE, an IEC NEMA body model was built geometrically following the NEMA specifications. The patient bed and the 700 mm-long cylindrical phantoms were also modelled. All phantom positions were set to replicate the experimental setup. Simulated data were collected over a single simulation of 270 s.

Acquired experimental and simulation data were reconstructed using the OSEM algorithm, with TOF information (TOFOSEM). Two iterations and 17 subsets were used with a 6.4-mm Gaussian filter in the transverse direction and a 3-point filter in the Z-axis (defined as standard). Scatter, random, attenuation, and normalisation corrections were performed. All reconstructed images had a size of 256 × 256 × 71 pixels, with a pixel size of 2.73 × 2.73 × 2.79 mm, covering a FOV of 700 mm. To assess the impact of corrections on the simulated data, an additional reconstruction, where the prompts were perfectly-corrected of random and scatter counts (i.e. only true counts were reconstructed), was performed and analysed.

The reconstructed images were evaluated in terms of contrast recovery coefficient (CRC), background variability (BV) and residual lung error (LE) as per NEMA guidelines. The image roughness (IR) [37] was also investigated.

### Electronics modelling/signal processing

**Fig. 2:**
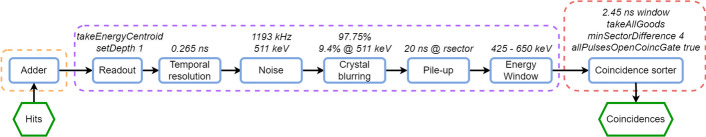
The complete digitiser model of the DMI 4-ring. In blue are the different modules with their associated parameters values. The orange dashed box encapsulates the processing of hits, the purple one the processing of pulses, and the last red box the processing of singles into coincidences

In GATE, the *digitiser* handles the signal processing of the photons, from photonic hits in crystals to coincidences. It consists of several modules that can be tuned through their associated parameters in order to accurately model the electronic chain of a PET system. Figure 2 shows the signal processing chain formed by the different modules of the DMI’s GATE model. Photonic hits are added into pulses, which are then processed into singles, to finally obtain coincidences.

The following sections describe the digitiser modules and the methods used to determine their optimal parameter values. The raw data acquired in “Scatter fraction, count losses, and randoms” were used to evaluate the system’s single, prompt and random rates over a wide activity range, between 0.8 and 31.2 kBq mL^−1^. For this purpose, experimental and simulated counts were obtained from list mode and sinograms, without additional data processing, to ensure proper initial modelling of the DMI in GATE.

#### Readout

After hits in a given volume are summed together into pulses by the adder module, pulses are then grouped by the readout module. The policy *takeEnergyCentroid* has been used with *setDepth 1*, positioning the summed pulses by weighting the crystal indices of each pulse by the deposited energy (Anger-logic scheme).

#### Energy and temporal windows/resolutions

The energy window, energy resolution, and coincidence timing window (CTW) parameter values have been set according to the manufacturer’s specifications [38]. The coincidence time resolution (CTR) parameter value has been set according to experiments performed on our DMI 4-ring, using the methodology proposed by Uribe et al. [39]. Using this method, a value of 375 ps was measured, which is in line with the values reported in the literature by other groups for the DMI 4-ring [26, 29, 40]. Energy thresholding is the final step in the signal processing of singles events and its lower and upper bounds were set at 425 keV and 650 keV, respectively. The energy resolution was set to 9.4% at 511 keV for all crystals. While the time resolution is usually defined in terms of CTR, GATE accepts only singles timing resolution (STR). As CTR is assumed to follow a Gaussian distribution, the associated FWHM can be used to find the STR as follows: STR = CTR/$\sqrt{2}$, giving STR = 0.265 ns. The coincidence window size in GATE represents the size of the window opened by an incoming single event. Since the nominal CTW value of the DMI system is $2\tau =4.9$ ns, the value $\tau =2.45$ ns was set in the digitiser.

#### Background noise and quantum efficiency

The background noise and quantum efficiency can be determined by following the method proposed by Salvadori et al. [41], using the experimental single counts *S*_exp_ acquired during the NEMA count losses test. These experimental counts were compared to simulated singles counts *S*_sim_ acquired under the same conditions, with simulation deadtime, background noise (Noise_sim_), and quantum efficiency (QE_sim_) ignored, i.e. removed from the digitiser. The low-activity data of both acquisitions (supposed to be unaffected by deadtime) were then used to fit a linear model according to (3), so as to determine QE_sim_ and Noise_sim_. Using this method, a background noise of 1193 kHz and a quantum efficiency of 97.75% were found for the DMI.

3 $$\begin{aligned} S_{\rm exp}= {\text{QE}}_{\rm sim} (S_{\rm sim} + {\text{Noise}}_{\rm sim}) \end{aligned}$$

#### Singles deadtime and pile-up

At high activity, count rates are impacted by two main non-linear effects: deadtime and pile-up. Photon detection losses induced by pile-up are linked to energy thresholding. On one hand, the stacking of two single events can move the signal of a photon within a true coincidence outside the energy window (diminishing the rate of true coincidences). On the other hand, it can also move the signal of a scattered photon inside the energy window (increasing the rate of scattered coincidences). Therefore, the pile-up value impacts the true-to-scatter ratio, and it must be modelled before energy discrimination. Deadtime can be modelled either before or after the energy window, with no subsequent impact on the true-to-scatter ratio. When modelled after the energy window, it enables the use of an experimentally determined value. If modelled before the energy window, an optimisation process is required.

Therefore, the balance between the deadtime and pile-up values is guided by the trues count rate provided by the GATE model. It can be determined using the NEMA count losses methodology applied to the simulated data used in section “Background noise and quantum efficiency”. For our model, the simulated true count rate was higher than the experimental true count rate, even at low activities. In this work, the pile-up value was determined through empirical optimisation, using the prompt count rates obtained during the NEMA count losses analysis. Simulations were performed by varying the pile-up values so as to obtain the best fit between experimental and simulated prompt rates, and a value of 20 ns was found to be optimal.

#### Coincidence sorting

After energy thresholding, the resulting singles are grouped into coincidences in a CTW of 4.9 ns (see “Energy and temporal windows/resolutions”). By setting the *allPulseOpenCoincGate* parameter to *true*, we ensured that each single opens its own coincidence window, whether or not a window is already opened. Multiple coincidences were handled using the *takeAllGoods* policy as this has been suggested to give the best coincidence matching to MC data for most PET system designs [42]. In addition, all coincidences had to satisfy a geometric condition to restrict coincidence formation within a given transaxial FOV. This is handled by the *minSectorDifference* parameter, which was set to four, resulting in a 70 cm transaxial FOV.

## Results

### Electronics modelling / Signal processing

**Fig. 3:**
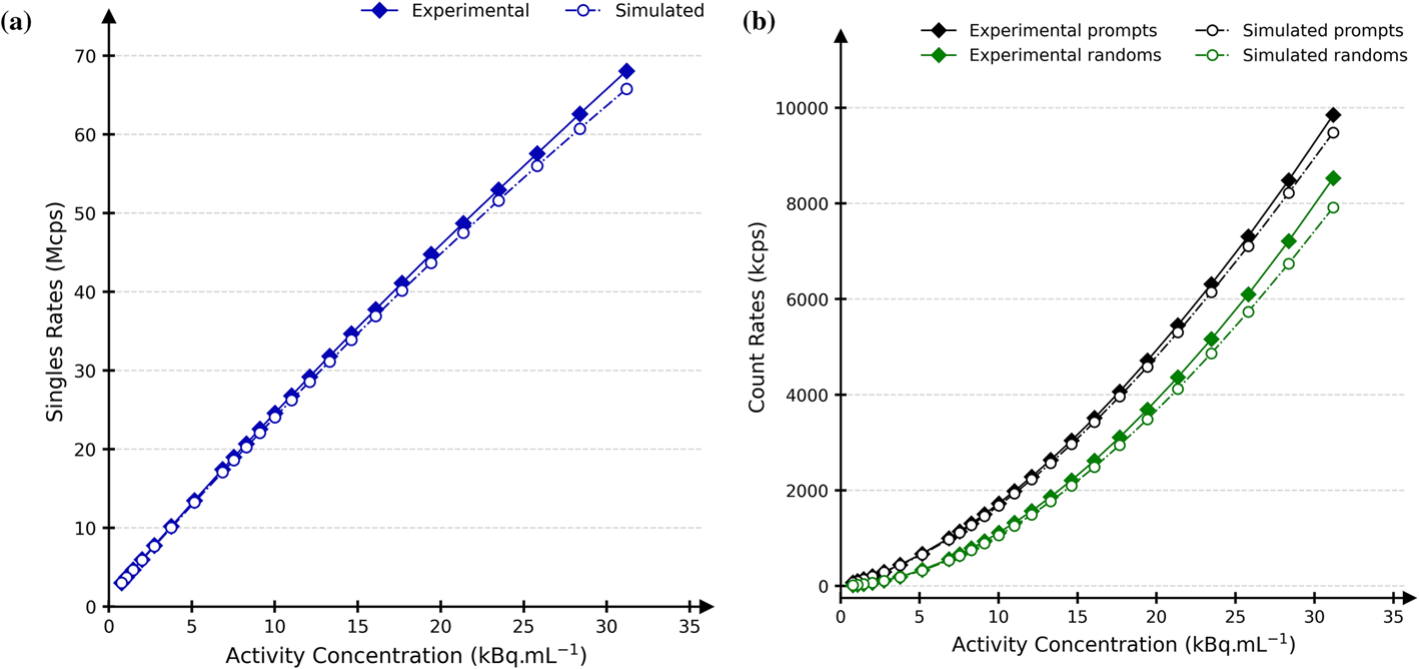
Singles rates in blue (**a**) and prompts (black) and randoms (green) rates (**b**) for simulated (dash-dotted) and measured (continuous) data

The system’s count rates of the DMI and its GATE model for average activity concentrations between 0.8 and 31.2 kBq mL^−1^ are shown in Fig. 3. The experimental single, prompt and random rates maximum relative differences were 2.0%, 2.5%, and 7.2%, respectively. This close relationship between the experimental and simulated global count rates provided a good basis for modelling, which was further investigated according to NEMA.

### Sensitivity

**Fig. 4:**
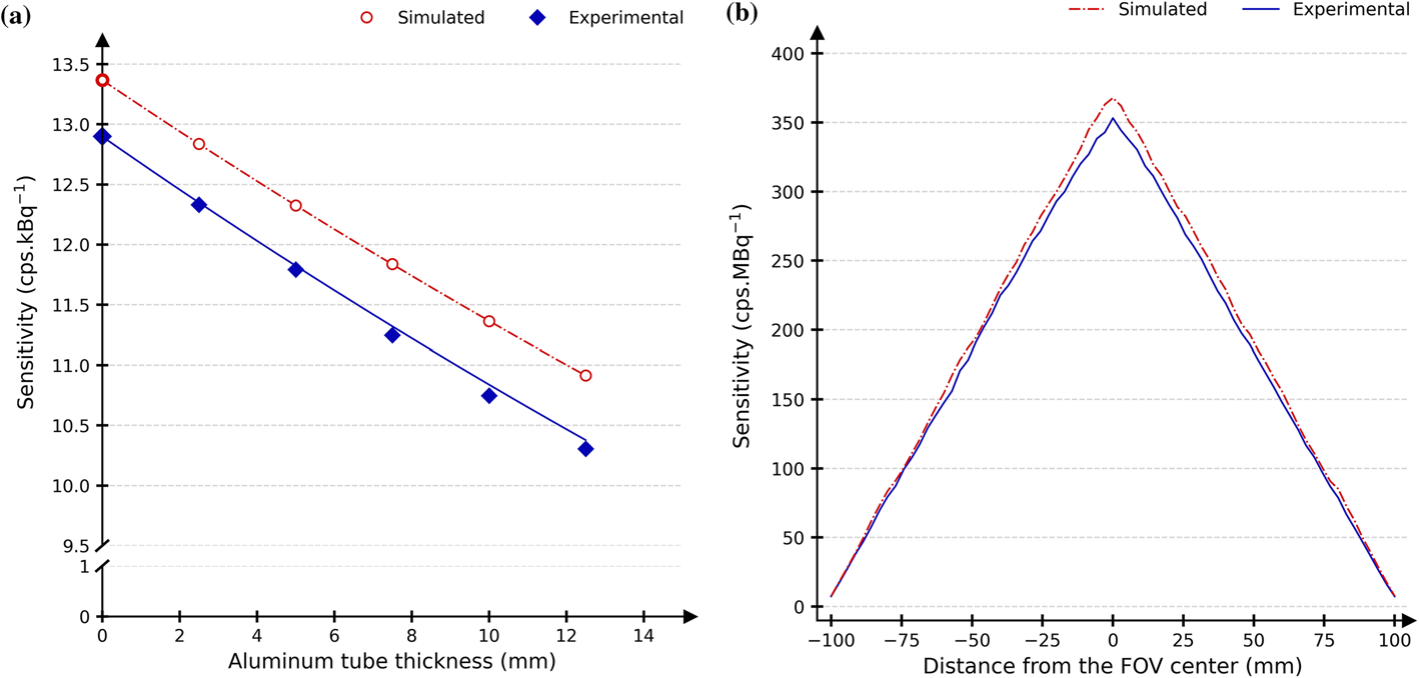
Report of **a** system sensitivity at the centre of the FOV for all aluminium thickness and the extrapolated sensitivity and **b** axial slices sensitivity with respect to the distance from FOV centre. Experimental (blue) and simulated (red) data are represented

Figure 4a shows the measured experimental and simulated sensitivities for all aluminium thicknesses as well as the extrapolated value obtained for zero absorber sensitivity. The experimental and simulated computed absorber-free sensitivities at the FOV centre were 12.90 cps/MBq and 13.37 cps/MBq, respectively, showing a 3.6% agreement. At 10 cm from the centre of the FOV, experimental and simulated sensitivities were 13.00 cps/MBq and 13.38  cps/MBq, respectively. Figure 4b shows a good agreement between experimental and simulated sensitivity profiles, where the largest relative difference for slice-wise sensitivity was 9.9%. Seven CPU days were needed for the simulation of this NEMA standard.

### Count rates and scatter fraction

**Fig. 5:**
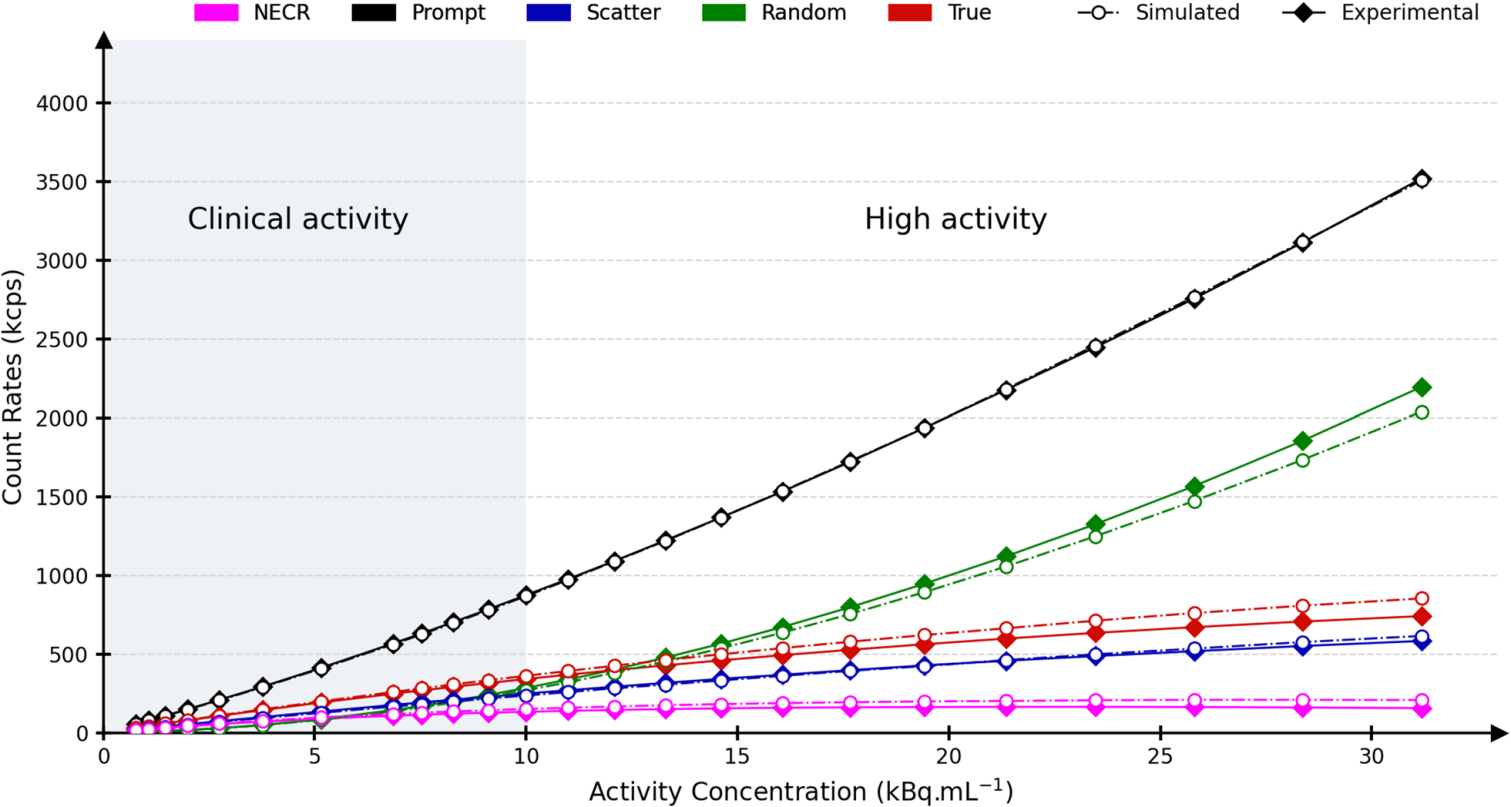
Prompt (black), random (green), scatter (blue) and true (red) count rates (kcps) for experimental (continuous) and simulated (dash-dotted) data relative to the activity concentration (kBq/mL). The noise equivalent count rates (NECR) are shown in purple. The clinical activity range (activity concentration < 10 kBq/mL) is outlined

**Fig. 6:**
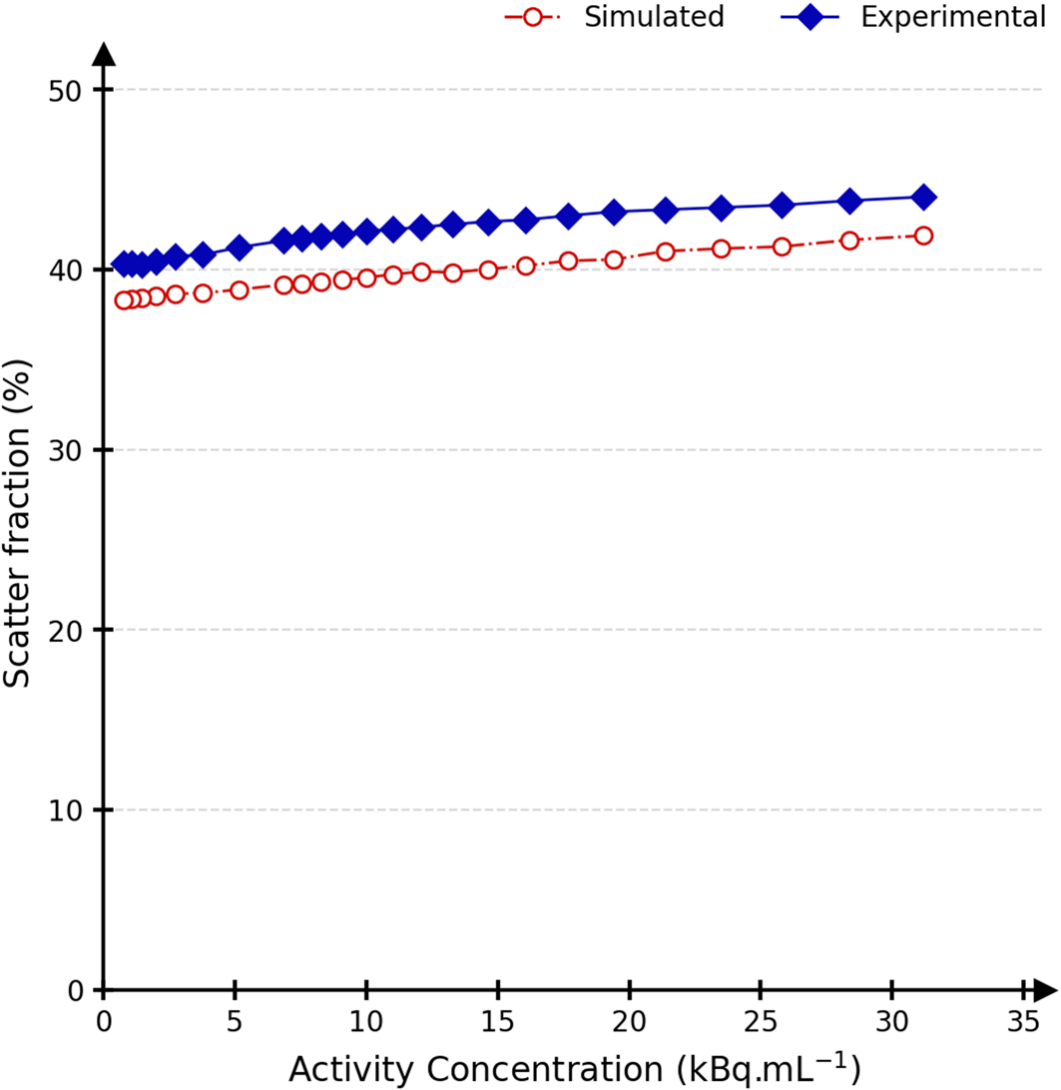
The scatter fraction (%) for experimental (continuous) and simulated (dash-dotted) data relative to the activity concentration (kBq/mL)

Figure 5 shows count rates computed according to the NEMA count losses test for experimental and simulated data. Prompt, random, scatter, and true coincidences rates maximum relative differences were 1.6%, 7.2%, 6.6%, and 15.1%, respectively, over the full activity range. When considering activities closer to the clinical activity concentrations (below 10 kBq/mL), the maximum relative difference for true count rates was 6.6%. The associated NEC rate curves are in a 33.1% agreement over the complete activity range, and in a 16.5% agreement within the clinical activity range. The peak noise equivalent count rates (NECR) was at 23.5 kBq/mL (164 kcps) and 25.8 kBq/mL (208 kcps) for experimental and simulated data, respectively. The scatter fraction is shown in Fig. 6, and its values at the peak NECR are 44.4% and 41.3% for experimental and simulated data, respectively. In total, 157 CPU days were needed to simulate the 24 frames with around ten million coincidences.

### Spatial resolution

Table 1 reports the spatial resolution in terms of FWHM and full width at tenth maximum (FWTM) for both simulated and experimental data, averaged over both axial positions (see Spatial resolution). The absolute differences ranged from 0.16 to 1.24 mm, and from 0.65 to 1.82 mm for the FWHM and FWTM, respectively. For all positions, the simulated spatial resolution values were found to be consistently smaller than their experimental counterparts. A total of 34 CPU days were used to run this spatial resolution simulation.

**Table 1:** Spatial resolution in terms of FWHM and FWTM for experimental and simulated data. For each profile, the difference between simulated and experimental data (in mm) is also reported

Profile direction	Experimental (mm)		Simulated (mm)		Difference (mm)	
	FWHM	FWTM	FWHM	FWTM	FWHM	FWTM
1 cm radial offset						
Radial	4.34	8.48	3.37	7.53	− 0.98	− 0.96
Tangential	4.17	8.20	3.64	7.47	− 0.54	− 0.73
Axial	4.49	10.07	4.25	8.26	− 0.24	− 1.82
10 cm radial offset						
Radial	5.66	11.30	5.18	9.97	− 0.49	− 1.37
Tangential	4.40	9.77	4.10	8.15	− 0.31	− 1.64
Axial	6.21	11.79	4.98	10.78	− 1.24	− 1.01
20 cm radial offset						
Radial	7.27	14.35	7.11	13.02	− 0.16	− 1.34
Tangential	5.10	9.88	4.67	9.21	− 0.43	− 0.68
Axial	6.29	12.45	5.82	11.81	− 0.47	− 0.65

Values are averaged over both axial positions (central transverse plane of the scanner and offset of three-eighths of the axial FOV from the FOV centre)

### Time-of-flight resolution

**Fig. 7:**
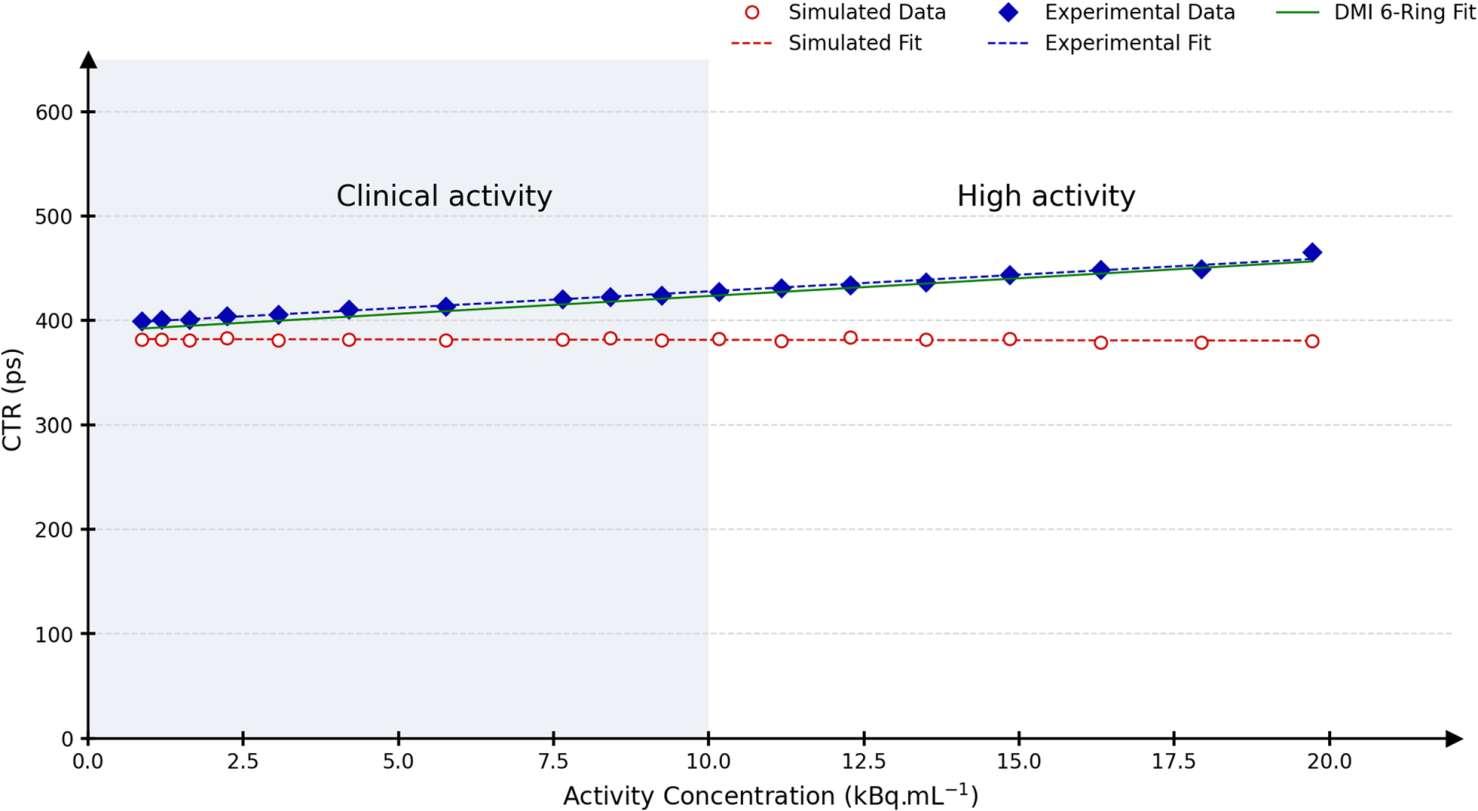
Experimental (blue) and simulated (red) CTR data points and the associated fits. The green fit is adapted from the time-of-flight resolution results presented by Zeimpekis et al. [43] for a DMI 6-ring scanner

Figure 7 shows the CTR as a function of the activity concentration *AC* for experimental and simulated data points, as well as a linear fit using these points. For comparison purposes, an additional linear fit is shown for a DMI 6-ring scanner (Gen2 system), adapted from Zeimpekis et al. [43]. The linear fit equations for the experimental DMI 4-ring, simulated DMI 4-ring and experimental DMI 6-ring are ${\text {CTR}}_{\rm cor}=3.19 AC + 395.9$, ${\text {CTR}}_{\rm cor}= -0.07 AC + 382.1$ and ${\text {CTR}}= 3.41AC + 389.2$, respectively. The experimental data shows a slight increase in CTR value with respect to the activity concentration, while the simulated CTR values are more stable across the activity range with an average value of 381.4 ps.

### Image quality

**Fig. 8:**
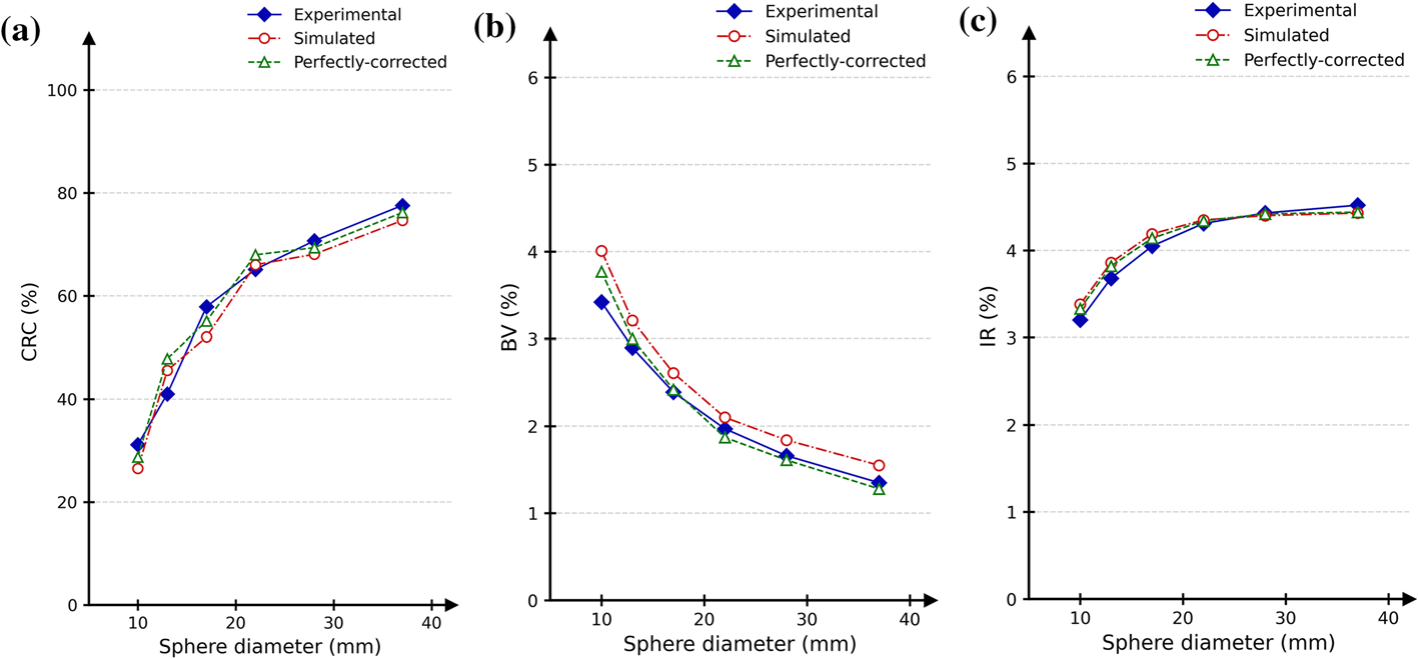
Comparison between experimental (blue continuous), simulated (red dash-dotted) and perfectly-corrected (green dashed) contrast recovery coefficient (**a)**, (**b**) background variability and (**c**) image roughness for the six hot spheres of the NEMA phantom/model

**Fig. 9:**
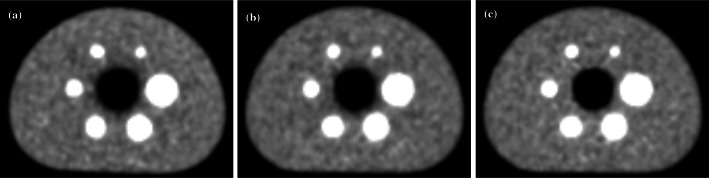
Visual comparison between the central slices of the TOFOSEM reconstruction of **a** experimental data, **b** simulated data with clinical-like corrections, and **c** simulated data with perfect corrections. All images were normalised by their maximum, and the same window and level were used

The CRC, BV and IR for experimental and simulated TOFOSEM reconstructions are shown in Fig. 8. The CRC for experimental and simulated reconstructions showed a maximum relative difference of 14.9% across all spheres, and a maximum relative difference of 5.7% was found when comparing IR. From the smallest to the largest sphere, the background variability ranged from 3.4 to 1.4%, and from 4.0 to 1.6% for the experimental and simulated reconstructions, respectively. The experimental LE was $11.4\%$ while the simulated LE was 7.5%. When comparing experimental and perfectly-corrected reconstructions, maximum relative differences of 17% and 10% were found on both CRC and BV, respectively. Differences of less than 5% were observed for the IR, and a LE of 5.4% was measured. A visual comparison of the central axial slice for experimental and simulated reconstructions is given in Fig. 9. This simulation required 161 days of CPU time.

## Discussion

We presented a complete Monte Carlo model for the DMI 4-ring. The model is able to mimic experimental count rates in a large activity range as well as generate clinical-like images through the use of a proprietary clinical package. During the development of the model, special care was taken to use only information that could be obtained from experimental data to allow realistic processing of the simulated data: to this end, only singles and prompts were extracted from the raw simulations in GATE. In addition, all sinogram data (experimental and simulated) were processed using the same processing pipeline, developed in Python/C++, and previously validated on experimental data with the manufacturer’s software tools (see Appendix “Simulation environment/framework”).

Our GATE model specifically takes into account the background noise of the scanner at low activity (i.e. mimic the natural radiation from LYSO crystals plus dark counts from electronic components) and the system count losses at high activity (deadtime and pile-up). Thanks to a specific method [7] to optimise these values within the digitiser, a good agreement was obtained in terms of singles (2.0%), prompts (2.5%), and random counts (7.2%) recorded in the sinograms, for activity concentrations up to 31.2 kBq/mL. This resulted in a 3.6% maximum relative difference between experimental and simulated data on system sensitivities. To recover inter-block Compton scatter, GE HealthCare systems use the Compton Scatter Recovery (CSR) method that is able to identify scatter events from neighbouring blocks and combine them into a true event to improve the sensitivity [40]. As this approach is not currently implemented in GATE, Khalif et al. [22] proposed to emulate the sensitivity gain of the CSR method by using the *TakeEnergyWinner* readout policy set at the highest level (*setDepth 1*) in the digitiser. Our model did not follow this approach as a very good agreement was obtained with the NEMA sensitivity and count rates tests using the *TakeEnergyCentroid* readout policy with *setDepth 1*.

Simulated prompt, random, true, and scatter rates, obtained from the NEMA count losses test, closely matched the experimental rates with maximum relative differences of 1.6%, 5.3%, 7.8%, and 6.6%, respectively, in a clinical range taken pejoratively of less than 10 kBq/mL on GE HealthCare systems [44]. At higher activity, true rates were shown to be in poorer agreement between experimental and simulated data, with an absolute relative difference of 15.1%. Therefore, our model does not provide the ability to improve the NEC rates at very high activity. This might also highlight the limitations of the current version of GATE’s digitiser, including its inability to emulate the CSR module.

The spatial resolution of our GATE model was found to have smaller nominal values for all sources compared to the DMI 4-ring values, where the differences between experimental and simulated FWHM were ranging from -0.16 to -1.24 mm. This trend is common for GATE-simulated PET [11, 12, 16, 22] as there is no digitiser module to enable spatial blurring for general PET scanner models. This spatial blurring arises from effects that are not properly accounted for in the simulation, such as Anger logic and crosstalk between crystals. This drawback of the digitiser is often corrected by applying a blur on the radial axis of the prompt and random sinograms [11, 16, 22] to obtain a closer match between experimental and simulated spatial resolutions.

Regarding the NEMA NU 2-2018 TOF resolution, we have investigated the experimental CTR as a function of activity (see Fig. 7) on our DMI 4-ring although this scanner is of an older generation (Gen1), prior to the NEMA NU 2-2018 report. As our in-house tool for this NEMA test could not be directly validated, a comparison is provided with Zeimpekis et al. [43], where the CTR (obtained using the manufacturer’s tool) is reported for a DMI 6-ring, a Gen2 system with an extended axial FOV. Besides, TOF data are not mashed on Gen2 systems. According to NEMA NU 2-2018, the obtained intercepts of CTR at 0 kBq mL$^{-1}$ for the DMI 4-ring Gen1 system and the DMI 6-ring Gen2 system were in a 1.8% agreement. Applying a correction factor for mashing allowed us to recover CTR$_{\rm cor}$ values relatively close to those obtained for Gen2 systems. The difference at 0 kBq mL$^{-1}$ (5.6%) between the experimental NEMA CTR$_{\rm cor}$ (395.9 ps) and the value obtained in “Energy and temporal windows/resolutions” (375 ps) might be due to the differences in the measurement methods. Regarding simulations, the CTR$_{\rm cor}$ was stable over the whole activity range, which is expected as there is no digitiser module in GATE to degrade the TOF resolution with respect to the singles rate. The simulated CTR$_{\rm cor}$ value differed by 1.8% from the value used in the digitiser. This was identified to come from the *takeEnergyCentroid* readout parameter, which relocates a given event at the centre of a crystal (after barycentric weighting according to Anger’s logic) but without updating the event timestamp. This resulted in an additional degradation of the TOF resolution equal to the time required to travel at most half the crystal thickness. On the other hand, the *takeEnergyWinner* parameter would return the exact spatial and temporal values of the interactions within the crystals, but does not correspond to the standard positioning scheme used in clinical PET scanners.

Our reconstruction framework is based on the manufacturer’s clinical software, enabling the use of similar reconstruction and correction methods between experimental and simulated data. Figure 9 shows a good visual similarity when comparing simulated and experimental data reconstructions. When using clinical-like corrections (normalisation, attenuation, random and scatter), CRC and BV were relatively close between experimental and simulated reconstructions. However, the trend for the simulated CRC as function of the sphere size is not as stable as the experimental one. This might be induced by the use of a single simulated image for the analysis, while three images were used for the experimental study. When investigating the perceived image noise in an image, IR is usually more appropriate than the NEMA BV metric, as IR measures the pixel-to-pixel variation rather than the homogeneity between regions [37]. Regarding IR, a better agreement (5.7%) was found between simulated and measured images. Although the reconstruction kernel is the same, small discrepancies are expected to be introduced by the reconstruction process due to the need to integrate specific GATE data to correct the simulated data for normalisation and attenuation. For example, the use of different attenuation maps could contribute to the observed differences in LE [25]. Furthermore, with perfect random and scatter corrections, CRC, BV and IR were found to be in good agreement with the experimental measurements (Fig. 9), indicating that some improvements could still be achieved when applying these two clinical-like corrections (RFS and model-based scatter) to simulated data. Finally, decay (during acquisition), well-counter, and deadtime corrections for simulated data have not yet been implemented in the current version of our framework. Therefore, for now, the model is able to produce realistic but not quantitative images in kBq/mL.

The performance characteristics of our DMI scanner available in our nuclear medicine department were similar to those reported in the literature [26, 27, 29]. Considering all four published sites, their results on a DMI-4 ring scanner differ from ours by $10.7\pm 4.4$% in peak NECR, $3.3\pm 3.1\%$ in sensitivity, and $-0.7\pm 8.3\%$ in volumetric resolution (FWHM) measured at 1-cm radial offset. Image quality results could not be directly compared as FOV, matrix size and reconstruction parameters were different between sites. Inter-system variance between published characteristics performance of the same scanner can be explained by differences in the manufacturing process, experimental uncertainties, and the use of different processing tools.

There are currently two DMI 4-ring GATE models in the literature. First, Tiwari et al. [23] focused on establishing a DMI 4-ring GATE model to then study virtual DMI models with extended axial FOVs, up to two meters. To evaluate their DMI 4-ring model, the NEMA sensitivity and count rates tests were simulated and compared with experimental results published in another study [26]. Reconstructions were performed using the STIR software only to evaluate the spatial resolution of their model. They found a good agreement between simulated and experimental results (less than 8% relative errors up to 25 kBq/mL for counting rates and 6.4% for sensitivity). The detailed configuration of their digitiser is not shown in this publication and cannot be compared with ours. However, differences can still be highlighted, such as the absence of background noise in their digitiser and the absence of the patient bed in their geometry. Some comparisons can also be inferred from their sensitivity and count rate results. Firstly, our modelled slice-based sensitivity profile does not exhibit any inter-ring offset, just like the experimental one. Secondly, the general shape of our simulated count rate curves is similar to that of the measured curves over the entire activity range and never crosses.

The second DMI 4-ring GATE model is proposed by Kalaitzidis et al. [25]. In their study, they focused on a pipeline to enable GATE data reconstruction using CASToR. They compared their simulated reconstructions with experimental data, reconstructed with both the PET toolbox and CASToR. Their GATE digitiser is detailed and is quite similar to ours in the clinical range except that they followed the approach given by Khalif et al. [22] to take into account the CSR method. The optimisation of their digitiser also leads them to different values of noise and detection efficiency. However, they did not consider deadtime effects in their model, preventing accurate simulation at high activity. Their IQ phantom was simulated outside NEMA NU 2-2018 specifications, i.e. without the NECR phantom abutting the IQ phantom, with a SBR of nine, and with a scan time of ten minutes. Chosen reconstruction parameters were different from ours. Their results are difficult to comment on because their graph of NEMA metrics (CRC, BV) versus sphere size is presented on a very compressed scale. Nevertheless, their simulated CRC seems to be close to the experimental one (within $10\%$) and their simulated BV also appears to be close when the experimental reconstruction is performed with CASToR, but relatively higher when performed with the PET Toolbox. The authors attribute the differences between CASToR and the PET Toolbox to the difference in axial filtering between the two reconstruction packages.

Our ultimate goal is to give a general method to generate realistic PET images from simulated data. The present GATE model is able to mimic the count rates of a DMI 4-ring scanner on a large scale of activities, which allows a certain margin for medical applications that require a high activity bolus administration [44]. Besides, we showed that reconstruction is possible through the manufacturer’s reconstruction toolbox, provided that adequate data is simulated to compute the corrections used by the manufacturer. The expected benefits of this methodology are the ability to use the same reconstruction kernel as the one used in clinic and to simplify the implementation of the different corrections required for quantification. While we recognise that this approach requires the ability to work with the proprietary package through a software license, we believe that reconstructions performed with our framework, rather than with third-party software, are more representative of the clinical reconstruction process. Finally, we are currently working on adapting the BSREM algorithm (Q.Clear) to simulated data.

## Conclusion

In this study, we built a full Monte Carlo model of the DMI 4-ring. Count rate capabilities (up to 10 kBq/mL) and sensitivities of the model were validated according to NEMA specifications. Reconstructions were performed by entering simulated data into the proprietary reconstruction package and resulted in a good agreement with the clinical reconstructions. This complete workflow (Monte Carlo model plus reconstruction framework) can be used to generate both perfectly-corrected and clinical-like images and optimise the imaging parameters in several clinical situations.

## Figures and Tables

**Fig. 10:**
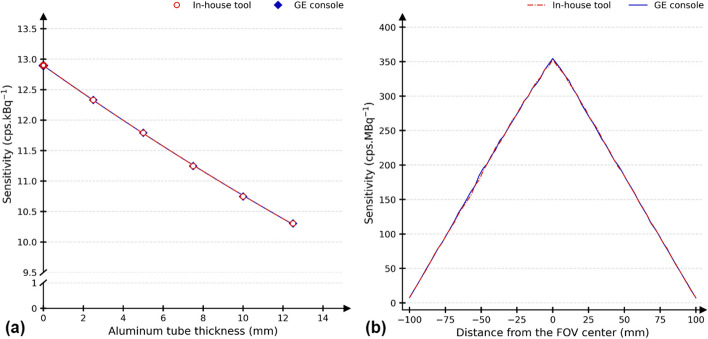
Validation of our in-house NEMA sensitivity tool (dash-dotted) against GE’s console (continuous). The extrapolated sensitivities for zero absorber (**a**) and the slice-wise sensitivities (**b**) were in a 1.0% and 4.9% agreement, respectively

**Fig. 11:**
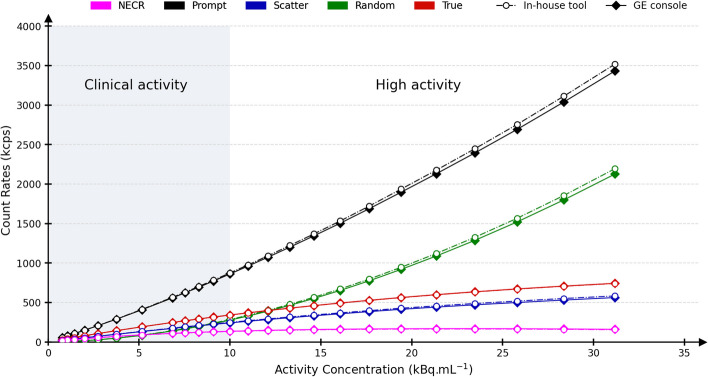
Validation of our NEMA count rates tool. Rates computed with our in-house tool (dash-dotted) were within a 3.6% margin of rates obtained using GE’s tool (continuous). This induced up to 2.2% relative difference for the scatter fraction

**Fig. 12:**
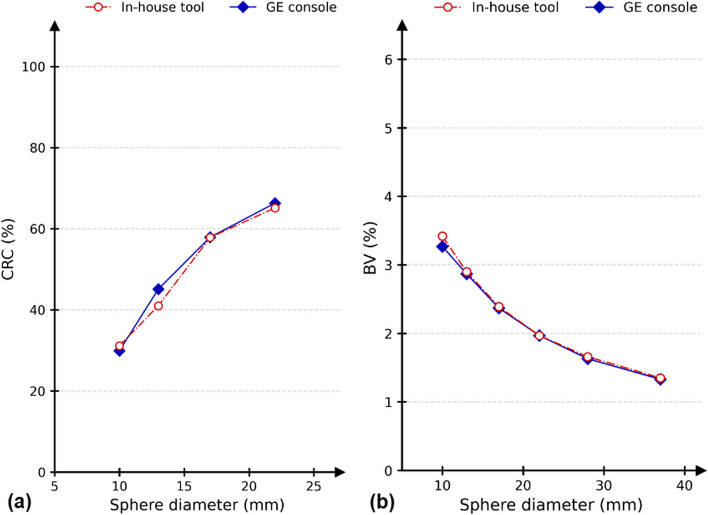
Validation of our in-house NEMA image quality tool. As the GE console is able to process only four hot spheres of the IEC phantom, **a** shows the CRC over these spheres, where a 9.1% agreement is found between the GE console (continuous) and our in-house tool (dash-dotted). The BV depicted in **b** was in a 4.8% agreement between both analyses

## Data Availability

The datasets used and/or analysed during this study are available from the corresponding author on reasonable request.

## Appendix

### Simulation environment/framework

In Figs. 10, 11, 12, and in Table 2 are reported the validation results of our in-house NEMA analysis software. For a given data set, the results obtained with the GE NEMA analysis tool (hereafter referred to as “GE console”) are compared against the results obtained using our in in-house tool, and the agreement between the obtain results is quantified by the maximum relative difference. The validation of our TOF resolution tool could not be performed since this NEMA test was not available in the GE console.

**Table 2:** Validation of our in-house NEMA spatial resolution tool

Profile direction	GE console (mm)		In-house tool (mm)		Relative difference (%)	
	FWHM	FWTM	FWHM	FWTM	FWHM (%)	FWTM (%)
1 cm radial offset						
Radial	4.47	8.86	4.34	8.48	− 2.9	− 4.3
Tangential	4.29	8.57	4.17	8.20	− 2.8	− 4.3
Axial	4.63	10.23	4.49	10.07	− 2.9	− 2.1
10 cm radial offset						
Radial	5.58	10.65	5.66	11.30	1.5	6.1
Tangential	4.53	9.76	4.40	9.77	− 2.8	0.2
Axial	6.23	11.98	6.21	11.79	− 1.0	− 1.6
20 cm radial offset						
Radial	7.40	14.14	7.27	14.35	− 1.8	1.5
Tangential	5.18	9.72	5.10	9.88	− 1.4	1.6
Axial	6.27	12.24	6.29	12.45	0.4	1.8

## Abbreviations

BV: Background variability
CCUB: Centre de Calcul de l’Université de Bourgogne
CRC: Contrast recovery coefficient
CSR: Compton scatter recovery
CT: Computed tomography
CTR: Coincidence time resolution
CTW: Coincidence timing window
DMI: Discovery MI
FBP: Filtered back-projection
FOV: Field-of-view
FWHM: Full width at half maximum
FWTM: Full width at tenth maximum
GATE: Geant4 application for tomographic emission
IQ: Image quality
IR: Image roughness
LE: Lung error
LYSO: Lutetium–Yttrium oxyorthosilicate
MC: Monte Carlo
NEC: Noise equivalent counts
NEMA: National Electrical Manufacturers Association
OSEM: Ordered-subsets expectation-maximisation
PET: Positron emission tomograpy
PSF: Point spread function
RFS: Randoms from singles
SBR: Sphere-to-background ratio
SF: Scatter fraction
SR: Spatial resolution
SSRB: Single-slice rebinning
STR: Singles timing resolution
TOF: Time-of-flight
